# A systematic review of comparative accuracy studies of the Kato-Katz and spontaneous sedimentation methods for schistosomiasis diagnosis

**DOI:** 10.1590/0037-8682-0335-2025

**Published:** 2026-04-17

**Authors:** Matheus Oliveira de Almeida, Rosa Camila Lucchetta, Sebastian Vernal, Carlos Graeff Teixeira

**Affiliations:** 1 Hospital Alemão Oswaldo Cruz, Responsabilidade Social, São Paulo, SP, Brasil.; 2 Universidade Federal do Espírito Santo, Departamento de Patologia e Núcleo de Doenças Infecciosas, Vitória, ES, Brasil.

**Keywords:** Schistosomiasis mansoni, Feces, Diagnosis

## Abstract

**Background:**

Although alternatives to the Kato-Katz (KK) method for the diagnosis of schistosomiasis are not widely available, other egg detection methods may have a role, especially considering the immediate need for confirmatory diagnosis and management of individual patients. One such widely available and easy-to-perform option is the Lutz method. We aimed to compare the diagnostic accuracy of KK and Lutz to support clinical decision-making.

**Methods:**

We conducted a systematic review searching PUBMED, EMBASE, and LILACS databases. We included primary diagnostic accuracy studies comparing KK and Lutz. No restriction was imposed on the type of test used as the reference standard. QUADAS-2 and QUADAS-C tools were used to assess the risk of bias and applicability, and GRADE was used to assess the certainty of evidence. The primary outcomes were sensitivity and specificity. Due to clinical and methodological heterogeneity, a meta-analysis was not performed.

**Results:**

Out of 1,060 citations, three studies were included in our review. The studies were cross-sectional and conducted in Brazil and Ethiopia. The sensitivity of Lutz ranged from 0.34 to 0.81, while that of KK ranged from 0.64 to 0.84. Specificity for both tests ranged from 0.81 to 1.0, with no significant differences observed between the diagnostic tests evaluated. Limitations included inconsistent reference standards, lack of blinding, and small sample sizes. The certainty of evidence ranged from very low to moderate.

**Conclusions:**

Data on the relative accuracy of the KK and Lutz methods for schistosomiasis diagnosis are limited. The current evidence is insufficient to determine the best method.

## INTRODUCTION

The Kato-Katz (KK) method is the recommended diagnostic tool for population screening of schistosomiasis^1^ ^,^ ^2^. Egg detection techniques provide confirmatory results with nearly 100% specificity due to the distinctive size and morphology of the eggs^1^ ^,^ ^2^. Katz et al.^3^ modified Kato’s thick smear to transform it into a quantitative approach, and estimates of infection intensity have become key for providing prognostic value. The number of eggs per gram (epg) is also used to classify areas as endemic based on infection severity (light, <100 epg; moderate, 101-399 epg; heavy, >400 epg)^4^.

The elimination of schistosomiasis as a public health issue has been defined as the presence of severe infection in less than 1% of a population^2^. Although the development of molecular and immunological methods is ongoing, the KK method remains the only affordable and standardized technique that provides the necessary quantitative estimate, especially when no other suitable screening tool is available or when egg burdens are still used to identify severe infection^1^ ^,^ ^2^ ^,^ ^5^ ^,^ ^6^.

Despite its many advantages, the KK method has reduced sensitivity for the detection of light *Schistosoma mansoni* infections, which predominate in many transmission areas, partly due to the success of control efforts^6^ ^-^ ^8^. The spontaneous sedimentation method, also known as the Lutz method (or the Hoffman, Pons, and Janer method)^9^ ^,^ ^10^, is another affordable and straightforward technique for the diagnosis of schistosomiasis in individual patients that can be performed in any field laboratory, especially when optimized sieving is used and large amounts of fecal sediment are examined^11^. To date, however, no systematic review has evaluated the relative accuracy of the Lutz and KK methods for the diagnosis of schistosomiasis. Thus, this systematic review was conducted to synthesize published data on the accuracy of the two methods to support ongoing expert discussions regarding schistosomiasis surveillance.

## METHODS

This systematic review was conducted according to the recommendations of the Cochrane Handbook for Systematic Reviews of Interventions and reported in accordance with PRISMA (Preferred Reporting Items for Systematic Reviews and Meta-Analyses)^12^ ^,^ ^13^. The protocol of the review was not prospectively registered.

### Eligibility criteria

We included primary studies assessing the relative diagnostic accuracy of the Lutz and KK methods for the clinical diagnosis of schistosomiasis in individual patients. No restriction was imposed on the type of test used as the reference standard, given the potential for an imperfect reference standard scenario^14^.

The primary outcomes were sensitivity and specificity, while the secondary outcomes were positive predictive value (PPV), negative predictive value (NPV), and egg recovery percentage (ERP). Cohort and cross-sectional studies with consecutive patient samples, as well as case-control studies and randomized clinical trials that provided sufficient data to estimate diagnostic accuracy, were eligible for inclusion in the review.

### Databases and search strategy

The following databases were searched from their inception until August 2024: MEDLINE and PubMed Central via PubMed, EMBASE, and LILACS. Search strategies are provided in Supplementary Material Table S1. 

### Study selection

Two independent evaluators (MOA and SV) performed study selection in two stages using the Rayyan platform^15^. First, the titles and abstracts of retrieved records were screened. Second, the full texts of studies deemed potentially eligible were accessed and evaluated. When necessary, a third reviewer (RL) resolved disagreements between the two reviewers.

Selection was not restricted by the language of the studies or the type of reference standard used, considering the usual absence of a perfect reference standard^14^, especially for egg detection methods with a low probability of yielding false-positive results but limited sensitivity for light infections.

### Data extraction

One reviewer (MOA) extracted the following information from the included publications: a) bibliographic data (main author, year of publication); b) study characteristics [study design, location, schistosomiasis endemicity level, known or estimated prevalence of schistosomiasis, funding source(s)]; c) sample characteristics (sex, age group, symptom duration); d) diagnostic protocol (numbers of fecal samples and slides examined); and e) accuracy-related data (numbers of true-positive, true-negative, false-positive, and false-negative results and/or other metrics supporting sensitivity, specificity, and accuracy calculations)^16^.

### Risk of bias assessment

We used the Quality Assessment of Diagnostic Accuracy Studies-2 (QUADAS-2) and the Quality Assessment of Diagnostic Accuracy Studies Comparative (QUADAS-C) to assess the risk of bias of the included studies. QUADAS-2 has four domains for risk of bias assessment (patient selection, index test, reference standard, flow & timing) and three domains related to applicability (patient selection, index test, reference standard). QUADAS-C has the same structure as QUADAS-2 but incorporates additional criteria for the comparison of index tests^17^ ^,^ ^18^.

### Data synthesis

The lack of clinical and methodological homogeneity across studies prevented the use of a meta-analysis with a random-effects model and multivariate method to obtain summary estimates of sensitivity and specificity. Thus, only descriptive results are provided here. Statistical heterogeneity was assessed through visual inspection of the sensitivity and specificity estimates, as well as the overlap of their confidence intervals (CI). I^2^ values were not calculated, as this statistic is not widely regarded as adequate for evaluating heterogeneity among accuracy studies^19^.

### Certainty assessment

The Grading of Recommendations, Assessment, Development, and Evaluation (GRADE) method was employed to determine the certainty of evidence for primary outcomes related to accuracy^20^. Each outcome was classified as having high, moderate, low, or very low certainty based on five domains: risk of bias, inconsistency, indirectness, imprecision, and publication bias. A minimally contextualized approach was used for the analysis, with thresholds for clinical relevance set at 0.7 for sensitivity and 0.9 for specificity. Due to the absence of established thresholds in the literature to define clinical relevance for the sensitivity and specificity of diagnostic tests for schistosomiasis, these thresholds were defined in consultation with a specialist in diagnostic tests for schistosomiasis.

## RESULTS

### Study selection

**FIGURE 1:**
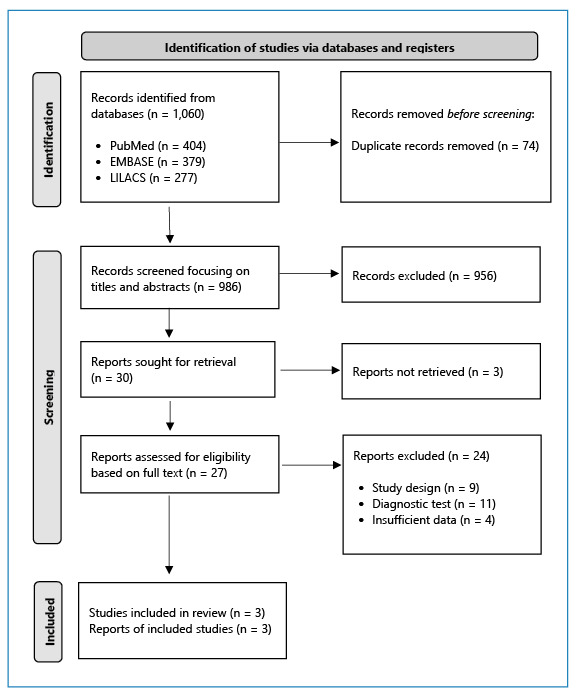
Flowchart of study selection process.

The search yielded 1,060 publications, of which 30 were selected for full-text analysis. Based on this assessment, three studies were included in the review (Figure 1). Reasons for exclusions after full-text assessment are summarized in Supplementary Material Table S2.

### Study characteristics

All three included studies had a cross-sectional design; two were conducted in Brazil and one was conducted in Ethiopia^21^ ^-^ ^23^. No information about funding, local endemicity, schistosomiasis prevalence, sex distribution, or symptom duration was provided in the studies. The studies included a total of 1,068 participants, with sample sizes varying from 217 individuals in Rabello et al^22^, 331 in Carvalho et al^21^ and 520 individuals in Fenta et al^23^. Age distributions in two of the studies were 6-14 years^23^, and 18-25 years^22^; Carvalho et al^21^ provided no information about included individuals’ age.

To assess Lutz and KK test performance, Carvalho et al.^21^ examined two slides each of three stool samples collected on alternate days. Rabello et al.^22^ examined two slides from each of one to six stool samples. No information on the test evaluation method was provided for the study conducted in Ethiopia^23^. Two included studies used a combination of the evaluated tests, including the Lutz and Kato-Katz tests^21^ ^,^ ^23^, as the reference standard. In the study by Rabello et al.^22^, the Lutz and Kato-Katz tests were compared with biopsy as the reference standard. Additional information on the evaluated test characteristics and diagnostic accuracy outcomes is provided in Table 1.

**TABLE 1:** Characteristics of evaluated tests and diagnostic accuracy data.

Study	Sample size	Index test	Quantity of slides and samples	Reference Standard	True-positive	False-negative	False-positive	True-negative
Fenta 2020	570	Lutz	-	Combination of tests (Lutz, Kato-Katz and Concentration in formalin-ether)	85	20	0	415
		Kato-Katz	-		67	38	0	415
Carvalho 2012	331	Lutz	2 slides of 3 samples on alternate days	Combination of tests (Lutz, Kato-Katz, Baermann-Moraes, Willis e *TF-Test*)	25	48	0	140
		Kato-Katz	2 slides of 3 samples on alternate days		54	19	0	140
Rabello 1992	217	Lutz	2 slides of 1 sample	Biopsy	54	33	9	121
		Kato-Katz	2 slides of 1 sample		60	27	9	121
		Lutz	2 slides of 2 samples		65	22	13	117
		Kato-Katz	2 slides of 2 samples		67	20	16	114
		Lutz	2 slides of 3 samples		70	17	20	110
		Kato-Katz	2 slides of 3 samples		73	14	19	111
		Lutz	2 slides of 4 samples		71	16	20	110
		Kato-Katz	2 slides of 4 samples		75	12	22	108
		Lutz	2 slides of 5 samples		71	16	23	107
		Kato-Katz	2 slides of 5 samples		77	10	22	108
		Lutz	2 slides of 6 samples		72	15	25	130
		Kato-Katz	2 slides of 6 samples		79	8	24	106

### Risk of bias in studies

None of the three included studies was judged to have a low risk of bias across the four domains assessed by both the QUADAS-2 and QUADAS-C tools. Bias-related limitations were identified in patient selection (QUADAS-C domain 1) for all three studies due to the lack of a paired and randomized controlled design, and in the reference standard (QUADAS-C domain 3) for the two studies in which combined test results were used. Details of the risk of bias assessment are provided in Supplementary Material Table S3 and Supplementary Material Figure S1.

### Results of individual studies and certainty of evidence

Effect estimates, along with 95% confidence intervals, for the outcomes of sensitivity, specificity, positive predictive value, and negative predictive value are provided in Table 2. None of the three included studies evaluated the outcome ‘percentage of egg recovery’.

**TABLE 2:** Results of sensitivity, specificity, positive predictive value, and negative predictive value of the included studies.

Study	Index test	Quantity of slides and samples	Reference Standard	Sensitivity (95% CI)	Specificity (95% CI)	PPV (95% CI)	NPV (95% CI)
Fenta 2020	Lutz	-	Combination of tests (Lutz, Kato-Katz and Concentration in formalin-ether)	**0.81 (0.72 - 0.87)**	**1 (0.99 - 1)**	1 (0.96 - 1)	0.95 (0.93 - 0.97)
	Kato-Katz	-		0.64 (0.54 - 0.72)	**1 (0.99 - 1)**	1 (0.95 - 1)	0.91 (0.89 - 0.94)
Carvalho 2012	Lutz	2 slides of 3 samples on alternate days	Combination of tests (Lutz, Kato-Katz, Baermann-Moraes, Willis e *TF-Test*)	0.34 (0.24 - 0.46)	**1 (0.97 - 1)**	1 (0.86 - 1)	0.74 (0.68 - 0.80)
	Kato-Katz	2 slides of 3 samples on alternate days		**0.74 (0.63 - 0.83)**	**1 (0.97 - 1)**	1 (0.93 - 1)	0.88 (0.82 - 0.93)
Rabello 1992	Lutz	2 slides of 1 sample	Biopsy	0.62 (0.52 - 0.72)	**0.93 (0.87 - 0.96)**	0.86 (0.75 - 0.93)	0.78 (0.71 - 0.85)
	Kato-Katz	2 slides of 1 sample		0.69 (0.59 - 0.78)	**0.93 (0.87 - 0.96)**	0.87 (0.77 - 0.94)	0.82 (0.75 - 0.88)
	Lutz	2 slides of 2 samples		**0.75 (0.65 - 0.83)**	**0.90 (0.84 - 0.94)**	0.83 (0.73 - 0.91)	0.84 (0.77 - 0.90)
	Kato-Katz	2 slides of 2 samples		**0.77 (0.67 - 0.85)**	0.88 (0.81 - 0.92)	0.81 (0.71 - 0.89)	0.85 (0.78 - 0.91)
	Lutz	2 slides of 3 samples		**0.80 (0.71 - 0.87)**	0.85 (0.77 - 0.90)	0.78 (0.68 - 0.86)	0.87 (0.79 - 0.92)
	Kato-Katz	2 slides of 3 samples		**0.84 (0.75 - 0.90)**	0.85 (0.78 - 0.90)	0.79 (0.70 - 0.87)	0.89 (0.82 - 0.94)
	Lutz	2 slides of 4 samples		**0.82 (0.72 - 0.88)**	0.85 (0.77 - 0.90)	0.78 (0.68 - 0.86)	0.87 (0.80 - 0.93)
	Kato-Katz	2 slides of 4 samples		**0.86 (0.77 - 0.92)**	0.83 (0.76 - 0.89)	0.77 (0.68 - 0.85)	0.90 (0.83 - 0.95)
	Lutz	2 slides of 5 samples		**0.82 (0.72 - 0.88)**	0.82 (0.75 - 0.88)	0.75 (0.65 - 0.84)	0.87 (0.80 - 0.92)
	Kato-Katz	2 slides of 5 samples		**0.89 (0.80 - 0.94)**	0.83 (0.76 - 0.89)	0.78 (0.68 - 0.86)	0.91 (0.85 - 0.96)
	Lutz	2 slides of 6 samples		**0.83 (0.73 - 0.89)**	0.81 (0.77 - 0.89)	0.74 (0.64 - 0.82)	0.90 (0.83 - 0.94)
	Kato-Katz	2 slides of 6 samples		**0.91 (0.83 - 0.95)**	0.81 (0.74 - 0.87)	0.77 (0.67 - 0.84)	0.93 (0.87 - 0.97)

Abbreviations: **CI:** confidence interval; **PPV:** positive predictive value; **NPV:** negative predictive value. Values in bold represent the estimates above of the threshold for clinical relevance.

### Sensitivity

The study conducted by Fenta et al.^23^, carried out with 570 participants, demonstrated a higher sensitivity, although not statistically significant, for the Lutz test (0.81; 95% CI 0.72 to 0.87) than for the KK test (0.64; 95% CI 0.54 to 0.72), with very low certainty of evidence. The study conducted by Carvalho et al.^21^ with 331 participants showed that the sensitivity of the Lutz test was significantly lower (0.34; 95% CI 0.24 to 0.46) than that of the KK test (0.74; 95% CI 0.63 to 0.83), with very low certainty of evidence. The reference standard used in these two studies was the combination of the evaluated test results.

The study conducted by Rabello et al.^22^ with 217 participants demonstrated, with moderate certainty of evidence, that the sensitivity of the Lutz and KK tests increases with the number of fecal samples used. The Lutz test presents lower sensitivity than the KK test, but the difference is not significant when compared with the reference test (biopsy).

### Specificity

Fenta et al.^23^ and Carvalho et al.^21^ reported specificity values for the Lutz and KK tests of 1.0 (95% CIs, 0.99-1 and 0.97-1.0, respectively), with very low certainty of evidence. Rabello et al.^22^ demonstrated that the specificity of both tests (Lutz and Kato-Katz), based on limited evidence, decreases with increasing numbers of fecal samples. There are no significant differences in specificity between the Lutz and Kato-Katz tests when compared with the reference standard (biopsy).

### Summary of findings

The sensitivity and specificity estimates, along with the absolute measures and assessments of the certainty of the evidence, are presented in Supplementary Material Tables S4 **,** S5, and S6 of the Supplementary Material. To create the summary of findings tables, a schistosomiasis prevalence of 4.3% with a 10% assumed variation was used^24^. The certainty of the evidence for sensitivity and specificity was generally very low, except in the study by Rabello et al.^22^, which yielded moderate certainty regarding the sensitivity of the Lutz (80%) and KK (84%) tests, based on the examination of two slides from each of three fecal samples per individual. Regarding specificity in Rabello et al.^22^, the estimates were based on low certainty of evidence, with the Lutz test at 81% and the Kato-Katz test at 85%.

## DISCUSSION

This systematic review included three studies comparing the diagnostic accuracy of the Lutz and KK methods for schistosomiasis. Overall, the available evidence on the performance of these tests is limited and variable. Both tests consistently displayed moderate to high specificity (0.81-1.0), whereas sensitivity estimates varied substantially, providing no clear evidence of the superiority of one method over the other. Methodological heterogeneity across studies, together with the very low to moderate certainty of evidence, precludes robust conclusions. These findings underscore the challenges of diagnosing schistosomiasis in low-endemicity settings and highlight the need for well-designed accuracy studies to guide clinical management and surveillance strategies.

The use of composite reference standards incorporating the index tests, as observed in Fenta et al.^23^ and Carvalho et al.^21^, may have introduced incorporation bias. This bias can potentially lead to overestimation of sensitivity and may also affect specificity estimates, although the direction and magnitude are less predictable^25^. In the absence of a perfect reference standard, latent class analysis may provide less biased estimates of diagnostic accuracy than composite reference standards by modelling the true disease status as a latent variable^26^.

The limited sensitivity of the KK test in low-endemicity settings is well established^7^ ^,^ ^11^ ^,^ ^27^. However, studies evaluating its accuracy for egg burdens of <100 epg are inadequate, leading to imprecise estimates, such as the reporting of a limit of detection of “between 100 and 50 epg.”^28^. Increasing the number of slides examined is one strategy to improve the detection of light infections, as demonstrated by Rabello et al.^22^ and other studies^29^ ^-^ ^31^. 

The KK method is inexpensive, straightforward, and quantitative, requiring no advanced equipment^3^ ^,^ ^32^. It is easy to learn, produces slides that can be stored for later examination, and is widely used by trained examiners globally. Currently, it remains the most effective quantitative technique for diagnosing intestinal schistosomiasis. However, it has some drawbacks beyond its limited sensitivity, including biosafety concerns related to unfixed faeces, limited capacity for detecting non-schistosomiasis intestinal parasites, and the potential distortion of delicate eggs due to the pressure required to spread the faecal material^33^ ^,^ ^34^. Furthermore, logistical constraints may arise from the unavailability of KK kits, particularly the plastic template designed to hold the standard 42 mg stool sample^35^.

The Lutz method is also an inexpensive and straightforward technique. Its main advantages include the absence of specialized equipment requirements and improved biosafety, as the feces are formalin-fixed. In addition, the use of fresh sediments enables the complete preservation of parasitic structures and shapes, including those of protozoan cysts and helminth eggs and larvae^9^. The latter is a good reason to use the Lutz test for the evaluation of individual patients with clinical signs attributable to intestinal parasitic infection. Although the Lutz method is not inherently quantitative, it can be used to estimate the numbers of eggs, larvae, and cysts when the initial amount of feces is known and the 10% sediment sampling volume to be examined is established. Its main disadvantages include a time-consuming and labour-intensive process, and the need to examine fresh spread preparations before they dry out.

Over the past five decades, numerous alternative diagnostic approaches have been developed, including immunological methods for the detection of antibodies (by serology) or antigens, and nucleic acid detection methods^6^. However, extensive evaluation is still needed before the widespread adoption of these techniques for routine population screening or individual diagnosis^36^ ^,^ ^37^. As suitable molecular or immunological alternatives are not yet fully available for populations in need, egg detection methods remain the standard option.

A potential recommendation to use the Lutz test as an alternative to the KK method for individual case investigation motivated this systematic review. Among the numerous publications identified, only three met the inclusion criteria, highlighting the need for well-designed accuracy studies, which are often a critical bottleneck in the implementation of new diagnostic tests.

The strengths of this review include a comprehensive and sensitive literature search and the application of rigorous methodological standards aligned with Cochrane Collaboration recommendations, including risk of bias and certainty of evidence assessments. This methodological rigor enhances the credibility and reliability of our findings. A limitation of this review is the lack of prospective registration of the review protocol. Another limitation is that data extraction was conducted by a single reviewer, potentially increasing the risk of data extraction errors and introducing bias.

An additional possible limitation is related to the thresholds for clinical relevance used to evaluate the sensitivity and specificity of the included studies. Due to the absence of established thresholds in the literature to define clinical relevance for the sensitivity and specificity of diagnostic tests for schistosomiasis, these thresholds were defined in consultation with a specialist in diagnostic tests for schistosomiasis. A sensitivity threshold of 70% was considered acceptable because both the Lutz and KK methods are primarily used for individual diagnosis, where test results are interpreted in conjunction with clinical and epidemiological information rather than in isolation. Conversely, a higher specificity threshold (90%) was adopted, as both methods rely on the identification of *Schistosoma mansoni* eggs, which exhibit distinctive morphology and are readily recognizable under light microscopy, facilitating high specificity. The application of these thresholds is essential to ensure the appropriate implementation of the GRADE approach for assessing the certainty of evidence in diagnostic accuracy estimates.

In conclusion, the evidence comparing the diagnostic accuracy of the Lutz and KK methods for schistosomiasis is limited and scarce, derived from only three studies at moderate to high risk of bias. Overall, the certainty of the evidence ranges from very low to moderate. With respect to sensitivity, the evidence is inconclusive regarding whether the Lutz test is better than the KK test. Regarding specificity, both tests demonstrated high specificity, showing no significant difference between them.
