# Supplementary material for: A systematic review of comparative accuracy studies of the Kato-Katz and spontaneous sedimentation methods for schistosomiasis diagnosis
Source: Rev Soc Bras Med Trop. 2026 Apr 17;59:e0335-2025. doi: 10.1590/0037-8682-0335-2025 (PMC13089450; doi:10.1590/0037-8682-0335-2025)
Supplement: Supplementary material [file 1678-9849-rsbmt-59-e0335-2025-md1.pdf]

SUPPLEMENTARY MATERIAL

Table S1. Search strategies in databases

Databases	Search Strategies	Number of references
PubMed	((("Schistosomiasis mansoni"[MeSH Terms] OR "schistosoma"[MeSH Terms] OR "schistosomiasis"[MeSH Terms] OR "schistosom*"[Title/Abstract] OR "schistosom* infection"[Title/Abstract] OR "bilharzia*"[Title/Abstract] OR "bilharzio*"[Title/Abstract] OR "snail fever"[Title/Abstract] OR "katayama fever"[Title/Abstract]) OR "schistosoma mansoni"[MeSH Terms]) OR "schistosoma mansoni"[Title/Abstract] OR "s mansoni"[Title/Abstract] OR "s mansoni"[Title/Abstract]) AND ("spontaneous sedimentation"[Title/Abstract] OR "spontaneous tube sedimentation"[Title/Abstract] OR "Lutz"[Title/Abstract] OR "HPJ"[Title/Abstract] OR "HH"[Title/Abstract] OR "Hoffman"[Title/Abstract] OR "Hoffman-Pons-Janer"[Title/Abstract] OR "Lutz/HPJ"[All Fields] OR "stool examination"[Title/Abstract] OR "parasitologic"[Title/Abstract] OR "coproscopic"[Title/Abstract]))	404
EMBASE	1- ('schistosomiasis mansoni'/exp OR 'schistosomiasis mansoni' OR 'schistosoma'/exp OR schistosoma OR 'schistosomiasis'/exp OR schistosomiasis OR schistosom*:ti,ab OR 'schistosom* infection':ti,ab OR bilharzia*:ti,ab OR bilharzio*:ti,ab OR 'snail fever':ti,ab OR 'katayama fever':ti,ab OR 'schistosoma mansoni'/exp OR 'schistosoma mansoni' OR 'schistosoma mansoni':ti,ab OR 's mansoni':ti,ab) AND ('spontaneous sedimentation' OR 'spontaneous tube sedimentation' OR lutz OR hoffman OR 'hoffman pons janer' OR 'stool examination'/exp OR 'stool examination' OR parasitologic OR coproscopic)<br>2- #1 AND [embase]/lim NOT ([embase]/lim AND [medline]/lim)	379
LILACS	(((mh:(esquistossomose)) OR (Bilharzíase) OR (Infecção por Schistosoma) OR (mh:(schistosoma mansoni)) OR (schistosomiasis mansoni) OR (schistosoma) OR (schistosomiasis)) AND ((sedimentación espontánea) OR (sedimentaçãoespontânea) OR (lutz) OR (hoffman, pons AND janer) OR (lutz/hpj) OR (hoffman) OR (spontaneous sedimentation) OR (spontaneous tube sedimentation) OR (examen de heces) OR (exame de fezes) OR (stool examination) OR (coproscopic) OR (coproscópico) OR (parasitological) OR (parasitológico)))	277
Total		1,060
