# Supplementary material for: A systematic review of comparative accuracy studies of the Kato-Katz and spontaneous sedimentation methods for schistosomiasis diagnosis
Source: Rev Soc Bras Med Trop. 2026 Apr 17;59:e0335-2025. doi: 10.1590/0037-8682-0335-2025 (PMC13089450; doi:10.1590/0037-8682-0335-2025)
Supplement: Supplementary material [file 1678-9849-rsbmt-59-e0335-2025-md2.pdf]

Table S2. List of excluded studies in full-text analysis selection phase, with the reasons for exclusion

Study	Title	Reason for exclusion
Amorim 2014	Schistosomias in the northern state of Espírito Santo, Brazil	Other study design
Carvalho 1996	Avaliação terapêutica do oltipraz na infecção humana pelo S. mansoni	Other study design
Coelho 2009	Use of a saline gradient for the diagnosis of schistosomiasis	Another diagnostic test
Coura 1974	Comparative study of the techniques of Lutz, Kato and Simoes Barbosa for the coprological diagnosis of Schistosomiasis mansoni	Insufficient data
Cunha 1987	Therapeutical evaluation of different dose regimens of praziquantel in Schistosomiasis mansoni, based on the quantitative oogram technique	Other study design
Domingues 1990	Reduction of morbidity in hepatosplenic schistosomiasis mansoni after treatment with praziquantel: a long term study	Other study design
Elsherif 2015	Long-term effect of mass chemotherapy of Schistosoma mansoni on infection rate and diagnosis accuracy	Another diagnostic test
Espírito-Santo 2013	Estudo comparativo da acurácia de diferentes técnicas para o diagnóstico laboratorial da esquistossomose mansoni em áreas de baixa endemicidade	Another diagnostic test
Espírito-Santo 2014a	Evaluation of real-time PCR assay to detect Schistosoma mansoni infections in a low endemic setting	Another diagnostic test
Espírito-Santo 2014b	Evaluation of the sensitivity of IgG and IgM ELISA in detecting Schistosoma mansoni infections in a low endemicity setting	Another diagnostic test
Espírito-Santo 2015	Comparative study of the accuracy of different technique for the laboratory diagnosis of schistosomiasis mansoni in areas of low endemicity in Barra Mansa City, Rio de Janeiro State, Brazil	Another diagnostic test
Fentahum 2021	Prevalence of Intestinal Parasites and Schistosoma mansoni and Associated Factors among Fishermen at Lake Tana, Northwest Ethiopia	Other study design
Ferreira 1984	Esquistossomose mansoni: diagnostico pela biopsia retal e parasitologico em 100 casos selecionados	Insufficient data
Gonçalvez 2006	Immunoassays as an auxiliary tool for the serodiagnosis of Schistosoma mansoni	Another diagnostic test

	infection in individuals with low intensity of egg elimination	
Guizelini 1987	Pesquisa de ovos “pesados” de helmintos nas fezes: estudo comparativo entre os métodos da sedimentação espontânea em água e de Ritchie	Other study design
Igreja 2007	Schistosoma mansoni-related morbidity in a low-prevalence area of Brazil: a comparison between egg excretors and seropositive non-excretors	Other study design
Igreja 2010	A 15-year follow-up study on schistosomiasis in a low-endemic area in Rio de Janeiro State, Brazil	Another diagnostic test
Lau 2023	Performance characteristics of diagnostic assays for schistosomiasis in Ontario, Canada	Another diagnostic test
Palmeira 2010	Prevalência da infecção pelo Schistosoma mansoni em dois municípios do Estado de Alagoas	Other study design
Pinheiro 2012	The combination of three faecal parasitological methods to improve the diagnosis of schistosomiasis mansoni in a low endemic setting in the state of Ceará, Brazil	Another diagnostic test
Pinto 1986	Avaliação da sensibilidade da técnica de eclosão de miracídios, de Suzuki, em comparação com outros processos empregados no diagnóstico da esquistossomose mansônica	Insufficient data
Soares 1995	Schistosomiasis in a low prevalence area: incomplete urbanization increasing risk of infection in Paracambi, RJ, Brazil	Other study design
Teixeira 2007	Detection of Schistosoma mansoni eggs in feces through their interaction with paramagnetic beads in a magnetic field	Another diagnostic test
Willcox 1991	The efficiency of Lutz, Kato-Katz and Baermann-Moraes (adapted) techniques association to the diagnosis of intestinal helminths	Insufficient data
