# Supplementary material for: A systematic review of comparative accuracy studies of the Kato-Katz and spontaneous sedimentation methods for schistosomiasis diagnosis
Source: Rev Soc Bras Med Trop. 2026 Apr 17;59:e0335-2025. doi: 10.1590/0037-8682-0335-2025 (PMC13089450; doi:10.1590/0037-8682-0335-2025)
Supplement: Supplementary material [file 1678-9849-rsbmt-59-e0335-2025-md3.pdf]

Table S3. Risk of bias assessment of included studies

Study	Tests	Risk of bias (QUADAS-2)				Applicability Concerns (QUADAS-2)			Risk of Bias (QUADAS-C)			
		**P**	**I**	**R**	**FT**	**P**	**I**	**R**	**P**	**I**	**R**	**FT**
Fenta, 2020	Lutz	Low	Unclear	High	Low	Low	Low	High	High	Low	High	Low
	Kato-Katz	Low	Unclear	High	Low	Low	Low	High				
Carvalho, 2012	Lutz	Low	Unclear	High	Low	Low	Low	High	High	Low	High	Low
	Kato-Katz	Low	Unclear	High	Low	Low	Low	High				
Rabello, 1992	Lutz	High	Unclear	Low	Low	Low	Low	Low	High	Low	Low	Low
	Kato-Katz	High	Unclear	Low	Low	Low	Low	Low				

Abbreviations: P: patient selection; I: index test; R: reference standard; FT: flow and timing
