# Supplementary material for: A systematic review of comparative accuracy studies of the Kato-Katz and spontaneous sedimentation methods for schistosomiasis diagnosis
Source: Rev Soc Bras Med Trop. 2026 Apr 17;59:e0335-2025. doi: 10.1590/0037-8682-0335-2025 (PMC13089450; doi:10.1590/0037-8682-0335-2025)
Supplement: Supplementary material [file 1678-9849-rsbmt-59-e0335-2025-md4.pdf]

Table S4. Summary of findings table of the comparison between index tests (Lutz e Kato-Katz) e reference standard (Concentration in formalin-ether diagnosing schistosomiasis

Sensitivity Kato-Katz	0.64 (95% CI: 0.54 to 0.72)
Specificity Kato-Katz	1.00 (95% CI: 0.99 to 1.00)

Sensitivity Lutz	0.81 (95% CI: 0.72 to 0.87)
Specificity Lutz	1.00 (95% CI: 0.99 to 1.00)

Prevalences	3.9%	4.3%	4.7%

Outcome*	№ of studies (№ of patients)	Study design	Factors that may decrease certainty of evidence					Effect per 1,000 patients tested			Test accuracy CoE
			Risk of bias	Indirectness	Inconsistency	Imprecision	Publication bias	Pre-test probability of 3.9%	Pre-test probability of 4.3%	Pre-test probability of 4.7%	
**True-positive with Lutz**	1 study[d] 520 patients	cross-sectional (cohort type accuracy study)	very serious[a]	serious[b]	not serious	not serious	none	32 (28 to 34)	35 (31 to 37)	38 (34 to 41)	⊕◯◯◯ Very low[a,b]
**False-negative with Lutz**								7 (5 to 11)	8 (6 to 12)	9 (6 to 13)	
**True-positive with Kato-Katz**			very serious[a]	serious[b]	not serious	serious[c]	none	25 (21 to 28)	28 (23 to 31)	30 (25 to 34)	⊕◯◯◯ Very low[a,b,c]
**False negative with Kato-Katz**								14 (11 to 18)	15 (12 to 20)	17 (13 to 22)	

<table>
<tr><th rowspan="2">Outcome*</th><th rowspan="2">№ of studies (№ of patients)</th><th rowspan="2">Study design</th><th colspan="5">Factors that may decrease certainty of evidence</th><th colspan="3">Effect per 1,000 patients tested</th><th rowspan="2">Test accuracy CoE</th></tr>
<tr><th>Risk of bias</th><th>Indirectness</th><th>Inconsistency</th><th>Imprecision</th><th>Publication bias</th><th>Pre-test probability of 3.9%</th><th>Pre-test probability of 4.3%</th><th>Pre-test probability of 4.7%</th></tr>
<tr><td>True negative with Lutz</td><td rowspan="4">1 study[d] 520 patients</td><td rowspan="4">cross-sectional (cohort type accuracy study)</td><td rowspan="2">very serious[a]</td><td rowspan="2">serious[b]</td><td rowspan="2">not serious</td><td rowspan="2">not serious</td><td rowspan="2">none</td><td>961 (951 to 961)</td><td>957 (947 to 957)</td><td>953 (943 to 953)</td><td rowspan="2">⨁◯◯◯ Very low[a,b]</td></tr>
<tr><td>False positive with Lutz</td><td>0 (0 to 10)</td><td>0 (0 to 10)</td><td>0 (0 to 10)</td></tr>
<tr><td>True-negative with Kato-Katz</td><td rowspan="2">very serious[a]</td><td rowspan="2">serious[b]</td><td rowspan="2">not serious</td><td rowspan="2">not serious</td><td rowspan="2">none</td><td>961 (951 to 961)</td><td>957 (947 to 957)</td><td>953 (943 to 953)</td><td rowspan="2">⨁◯◯◯ Very low[a,b]</td></tr>
<tr><td>False-positive with Kato-Katz</td><td>0 (0 to 10)</td><td>0 (0 to 10)</td><td>0 (0 to 10)</td></tr>
</table>

Explanations:

a. The study was considered at high risk of bias for more than one domain (patient selection and reference standard) of the QUADAS-C tool.

b. The reference standard used was inappropriate, as the results of the evaluated tests were combined.

c. The upper limit of the confidence interval crosses the clinical relevance threshold for sensitivity.

d. Study of Fenta et al. (2020)

* True-positive (patients with schistosomiasis); False-negative (patients incorrectly classified as not having schistosomiasis); False-negative (patients incorrectly classified as not having schistosomiasis); True-negative (patients without schistosomiasis).

Abbreviations: CI: confidence of interval; CoE: certainty of evidence.
