# Supplementary material for: A systematic review of comparative accuracy studies of the Kato-Katz and spontaneous sedimentation methods for schistosomiasis diagnosis
Source: Rev Soc Bras Med Trop. 2026 Apr 17;59:e0335-2025. doi: 10.1590/0037-8682-0335-2025 (PMC13089450; doi:10.1590/0037-8682-0335-2025)
Supplement: Supplementary material [file 1678-9849-rsbmt-59-e0335-2025-md5.pdf]

Table S5. Summary of findings table of the comparison between index tests (Lutz e Kato-Katz) and reference standard (diagnosing schistosomiasis

Sensitivity Kato-Katz	0.74 (95% CI: 0.63 to 0.83)
Specificity Kato-Katz	1.00 (95% CI: 0.97 to 1.00)

Sensitivity Lutz	0.34 (95% CI: 0.24 to 0,46)
Specificity Lutz	1.00 (95% CI: 0,99 to 1,00)

Prevalences	3.9%	4.3%	4.7%

Outcome*	№ of studies (№ of patients)	Study design	Factors that may decrease certainty of evidence					Effect per 1,000 patients tested			Test accuracy CoE
			Risk of bias	Indirectness	Inconsistency	Imprecision	Publication bias	Pre-test probability of 3.9%	Pre-test probability of 4.3%	Pre-test probability of 4.7%	
**True-positive with Lutz**	1 study[d] 331 patients	cross-sectional (cohort type accuracy study)	very serious[a]	serious[b]	not serious	not serious	none	13 (9 to 18)	15 (10 to 20)	16 (11 to 22)	⊕◯◯◯ Very low[a,b]
**False-negative with Lutz**								26 (21 to 30)	28 (23 to 33)	31 (25 to 36)	
**True-positive with Kato-Katz**			very serious[a]	serious[b]	not serious	serious[c]	none	29 (25 to 32)	32 (27 to 36)	35 (30 to 39)	⊕◯◯◯ Very low[a,b,c]
**False negative with Kato-Katz**								10 (7 to 14)	11 (7 to 16)	12 (8 to 17)	
**True negative with Lutz**	1 study[d] 331 patients	cross-sectional (cohort	very serious[a]	serious[b]	not serious	not serious	none	961 (932 to 961)	957 (928 to 957)	953 (924 to 953)	

Outcome*	№ of studies (№ of patients)	Study design	Factors that may decrease certainty of evidence					Effect per 1,000 patients tested			Test accuracy CoE
			Risk of bias	Indirectness	Inconsistency	Imprecision	Publication bias	Pre-test probability of 3.9%	Pre-test probability of 4.3%	Pre-test probability of 4.7%	
**False positive with Lutz**		type accuracy study)						0 (0 to 29)	0 (0 to 29)	0 (0 to 29)	⊕○○○ Very low[a,b]
**True-negative with Kato-Katz**			very serious[a]	serious[b]	not serious	not serious	none	961 (932 to 961)	957 (928 to 957)	953 (924 to 953)	⊕○○○ Very low[a,b]
**False-positive with Kato-Katz**								0 (0 to 29)	0 (0 to 29)	0 (0 to 29)	

Explanations:

a. The study was considered at high risk of bias for more than one domain (patient selection and reference standard test) of the QUADAS-C tool.

b. The reference test used was inappropriate, as the results of the evaluated tests were combined.

c. The upper limit of the confidence interval crosses the clinical relevance threshold for sensitivity.

d. Study of Carvalho et al (2012)

* True-positive (patients with schistosomiasis); False-negative (patients incorrectly classified as not having schistosomiasis); False-negative (patients incorrectly classified as not having schistosomiasis); True-negative (patients without schistosomiasis).

Abbreviations: CI: confidence of interval; CoE: certainty of evidence.
