# Supplementary material for: A systematic review of comparative accuracy studies of the Kato-Katz and spontaneous sedimentation methods for schistosomiasis diagnosis
Source: Rev Soc Bras Med Trop. 2026 Apr 17;59:e0335-2025. doi: 10.1590/0037-8682-0335-2025 (PMC13089450; doi:10.1590/0037-8682-0335-2025)
Supplement: Supplementary material [file 1678-9849-rsbmt-59-e0335-2025-md6.pdf]

Table S6. Summary of findings table of the comparison between index tests (Lutz e Kato-Katz*) and reference standard (biopsy) diagnosing schistosomiasis

Sensitivity Kato-Katz	0.84 (95% CI: 0.75 to 0.90)
Specificity Kato-Katz	0.85 (95% CI: 0.78 to 0.90)

Sensitivity Lutz	0.80 (95% CI: 0.71 to 0.87)
Specificity Lutz	0.81 (95% CI: 0.77 to 0.90)

Prevalences	3.9%	4.3%	4.7%

Outcome**	№ of studies (№ of patients)	Study design	Factors that may decrease certainty of evidence					Effect per 1,000 patients tested			Test accuracy CoE
			Risk of bias	Indirectness	Inconsistency	Imprecision	Publication bias	Pre-test probability of 3.9%	Pre-test probability of 4.3%	Pre-test probability of 4.7%	
**True-positive with Lutz**	1 study[c] 217 patients	cross-sectional (cohort type accuracy study)	very serious[a]	not serious	not serious	not serious	none	31 (28 to 34)	34 (31 to 37)	38 (33 to 41)	⊕⊕⊕◯ Moderate[a]
**False-negative with Lutz**								8 (5 to 11)	9 (6 to 12)	9 (6 to 14)	
**True-positive with Kato-Katz**			very serious[a]	not serious	not serious	not serious	none	33 (29 to 35)	36 (32 to 39)	39 (35 to 42)	⊕⊕⊕◯ Moderate[a]
**False negative with Kato-Katz**								6 (4 to 10)	7 (4 to 11)	8 (5 to 12)	

Outcome**	№ of studies (№ of patients)	Study design	Factors that may decrease certainty of evidence					Effect per 1,000 patients tested			Test accuracy CoE
			Risk of bias	Indirectness	Inconsistency	Imprecision	Publication bias	Pre-test probability of 3.9%	Pre-test probability of 4.3%	Pre-test probability of 4.7%	
**True negative with Lutz**	1 study[c] 217 patients	cross-sectional (cohort type accuracy study)	very serious[a]	not serious	not serious	serious[b]	none	817 (740 to 865)	813 (737 to 861)	810 (734 to 858)	⊕⊕◯◯ Low[a,b]
**False positive with Lutz**								144 (96 to 221)	144 (96 to 220)	143 (95 to 219)	
**True-negative with Kato-Katz**			very serious[a]	not serious	not serious	serious[b]	none	817 (750 to 865)	813 (746 to 861)	810 (743 to 858)	⊕⊕◯◯ Low[a,b]
**False-positive with Kato-Katz**								144 (96 to 211)	144 (96 to 211)	143 (95 to 210)	

Explanations:

a. The study was considered at high risk of bias for one domain (patient selection) of the QUADAS-C tool.

b. The upper limit of the confidence interval crosses the clinical relevance threshold for specificity.

c. Study of Rabello et al (1992)

* The data refer to the results of the Lutz and Kato-Katz tests performed on 2 slides of 3 samples

** True-positive (patients with schistosomiasis); False-negative (patients incorrectly classified as not having schistosomiasis); False-negative (patients incorrectly classified as not having schistosomiasis); True-negative (patients without schistosomiasis).

Abbreviations: CI: confidence of interval; CoE: certainty of evidence.
