# Supplementary figures and images for: A systematic review of comparative accuracy studies of the Kato-Katz and spontaneous sedimentation methods for schistosomiasis diagnosis
Source: Rev Soc Bras Med Trop. 2026 Apr 17;59:e0335-2025. doi: 10.1590/0037-8682-0335-2025 (PMC13089450; doi:10.1590/0037-8682-0335-2025)

**Figure S1.** Summary of risk of bias assessment of the included studies

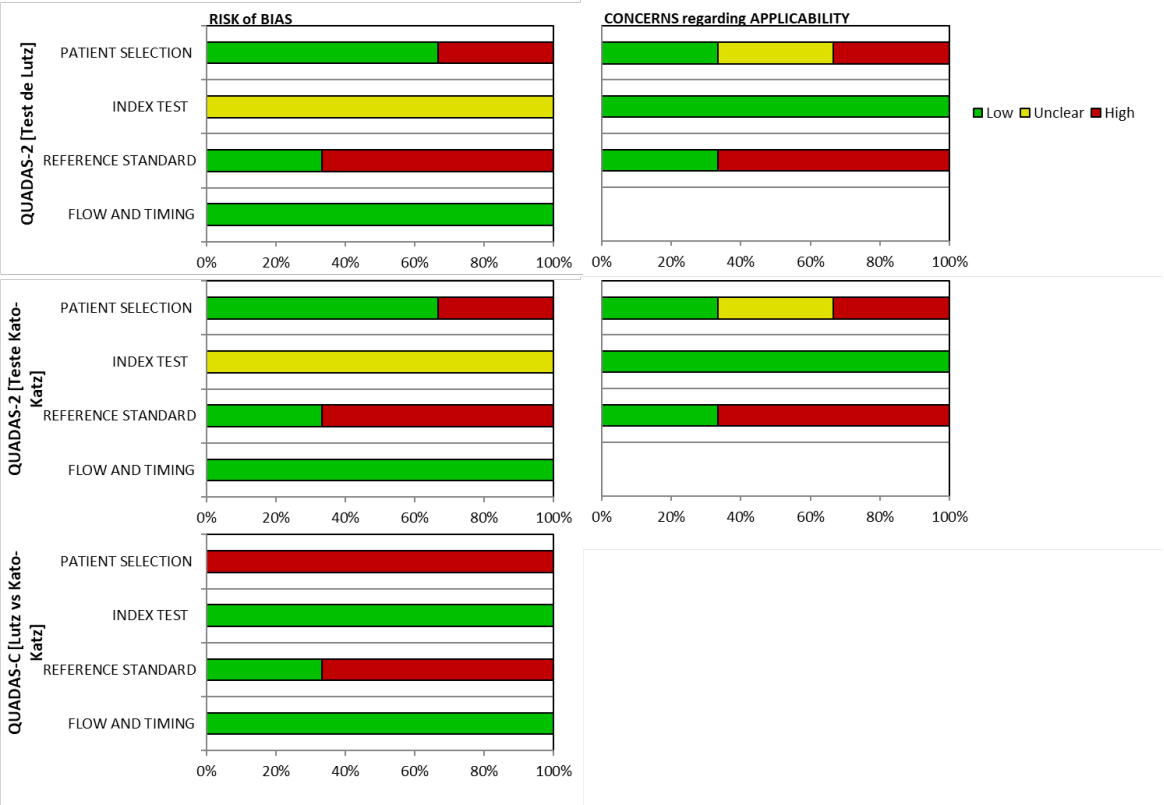

Supplement: Supplementary material [file 1678-9849-rsbmt-59-e0335-2025-md7.pdf]
